# The Anti-tumor Effects of *p-*Coumaric Acid on Melanoma A375 and B16 Cells

**DOI:** 10.3389/fonc.2020.558414

**Published:** 2020-10-16

**Authors:** Xue Hu, Zihui Yang, Wenjing Liu, Zhaohai Pan, Xin Zhang, Minjing Li, Xiaona Liu, Qiusheng Zheng, Defang Li

**Affiliations:** ^1^Yantai Key Laboratory of Pharmacology of Traditional Chinese Medicine in Tumor Metabolism, School of Integrated Traditional Chinese and Western Medicine, Binzhou Medical University, Yantai, China; ^2^Key Laboratory of Xinjiang Endemic Phytomedicine Resources of Ministry of Education, School of Pharmacy, Shihezi University, Shihezi, China

**Keywords:** *p-*coumaric acid (*p-*CA), melanoma, B16 cells, A375 cells, apoptosis, cell cycle arrest

## Abstract

**Background:** Existing research shows that *p-*coumaric acid (*p-*CA) can inhibit the proliferation of a variety of tumor cells *in vitro*. However, there are no reports on the anti-tumor effects of *p-*CA on melanoma cells. In this study, the inhibitory effects of *p-*CA on mouse melanoma B16 and human melanoma A375 cells are reported, and the related mechanisms are investigated.

**Methods:** CCK-8 assay was used to detect the effects of *p-*CA on cell vitality, colony formation assay was used to observe the effects on cell proliferation, Hoechst 33,258 staining was used to observe the morphology of apoptotic cells, flow cytometry was used to detect the effects on apoptosis and the cell cycle, and western blot was used to measure the levels of cell cycle- and apoptosis-related signaling pathway proteins.

**Results:** *p-*CA significantly inhibits cell proliferation of A375 and B16 cells in a dose-dependent manner and obviously induced cell morphological changes. *p-*CA arrested A375 cells in the S phase by downregulating the cell cycle-related proteins Cyclin A and CDK2, and arrested B16 cells in the G0–G1 phase through downregulating the cell cycle-related proteins Cyclin E and CDK2. In addition, *p-*CA significantly promoted apoptosis of A375 and B16 cells. Furthermore, *p-*CA significantly upregulated the levels of Apaf1 and Bax and downregulated the levels of Bcl-2, and subsequently increased the levels of cytoplasmic cytochrome c (Cyto-c), cleaved caspase-3, and cleaved caspase-9, leading to apoptosis in A375 and B16 cells.

**Conclusion:*** p-*CA can significantly inhibit the proliferation of human and mouse melanoma cells *in vitro*. Our research is a step in the development of anti-melanoma drugs.

## Introduction

Malignant melanoma (MM) is a malignant tumor of melanocytes originating from the neural crest, which has a high degree of malignancy, easily spreads, and has a poor prognosis. It is one of the most common tumors in the world, and in recent years, its incidence has increased each year. Melanoma is most common in Caucasian people, but its incidence has increased in recent years among Asians. In addition, unlike other solid tumors, melanoma mainly affects young and middle-aged people (the median age at the time of diagnosis is 57); the incidence increases linearly between the ages of 25 and 50 and then decreases (1). Therefore, melanoma is a big threat that seriously affects the quality of human life.

The most common treatment for melanoma is surgical resection. Unfortunately, this treatment can only be given in the early stage of local malignancy. The metastatic stage of malignant melanoma still presents huge treatment challenges (2). At present, targeted therapy and immunotherapy are the main treatments for metastatic melanoma, and they have become the focus of clinical research. Although some positive results have been achieved for targeted therapy and immunotherapy at this stage, these treatment methods can only be applied in a narrow population, are expensive, and require a high medical level, and can therefore not be widely used in clinical practice.

*p-*Coumaric acid (*p*-CA), also known as 4-hydroxycinamic acid, has a molecular formula of C_9_H_8_O_3_. *p-*CA is a phenolic acid that is non-toxic to mice (LD_50_ = 2,850 mg·kg^−1^) (3). It is widely found in mushrooms, grains (corn, rice, oats, and wheat), fruits (apples, pears, and grapes), and vegetables (carrots, potatoes, beans, onions, and tomatoes) (4) and has anti-inflammatory (5), antioxidant (4), anti-diabetic (6), anti-platelet aggregation (3), and anti-cancer (7) effects, among other biological functions. Recent studies have shown that *p*-CA has the ability to inhibit the proliferation and migration of tumor cells; for example, it can significantly inhibit the proliferation and migration of human lung cancer A549 cells and colon adenocarcinoma HT29-D4 cells in a dose-dependent manner (8). *p-*CA can also promote apoptosis of tumor cells; for example, *p-*CA inhibits the formation of polyps by improving the detoxification and apoptotic effects of 1,2-dimethylhydrazine on the rat colon (9).

Other pharmacological effects of *p*-CA include the inhibition of melanin production. Tyrosinase (TYP) is a key enzyme that catalyzes the production of melanin. The structure of *p-*CA is similar to that of tyrosine (TYP), and both compete for the active site on TYP (10). *p*-CA inhibits TYP activity, thereby inhibiting the formation of melanin in the cells.

In this study, we investigated the effects of *p-*CA on the proliferation of human melanoma A375 cells and mouse melanoma B16 cells. The results showed that *p-*CA can inhibit the proliferation of melanoma cells by causing cell cycle arrest and inducing apoptosis.

## Results

### *p*-CA Inhibits Proliferation and Colony Formation in A375 and B16 Cells

**Figure 1:**
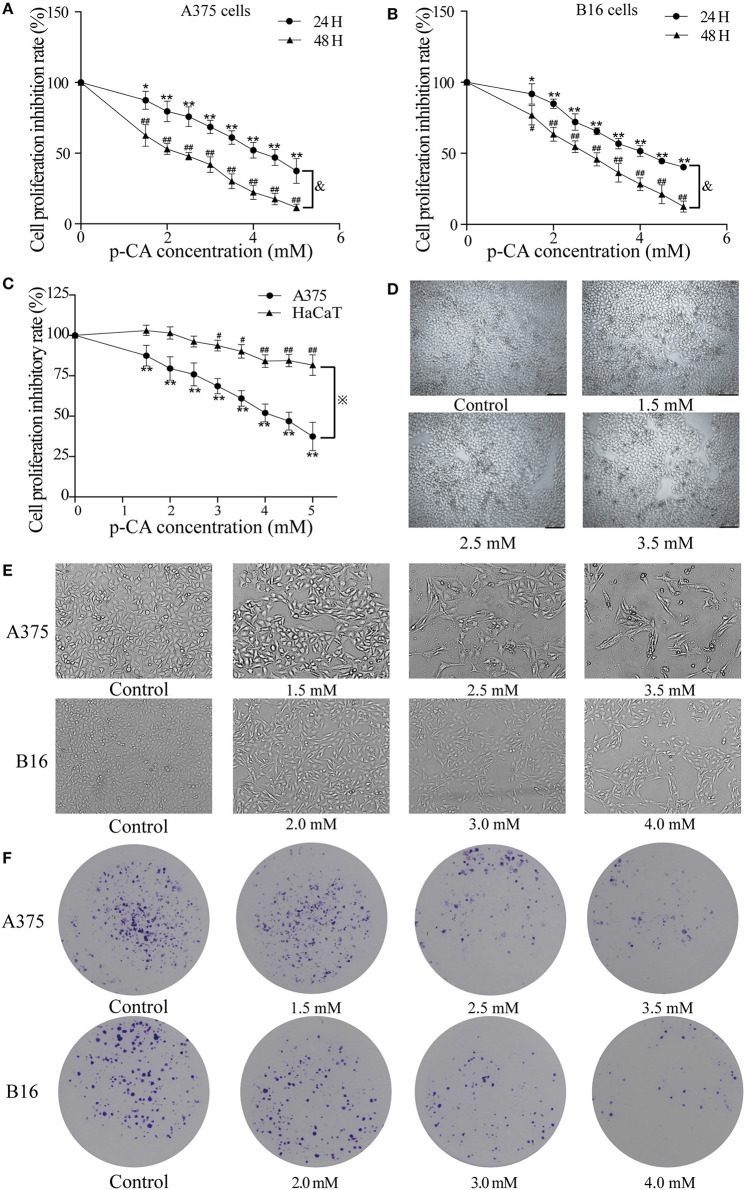
*p*-CA inhibits proliferation and colony formation of A375 and B16 cells. **(A,B)** A375 and B16 cells were treated with p-CA. After incubation for 24 or 48 h, the proliferation rates of A375 and B16 cells were measured by CCK-8 assay. **(C)** HaCaT, A375 and B16 cells were treated with *p*-CA. After incubation for 24 h, the proliferation rates of HaCaT, A375 and B16 cells were measured by CCK-8 assay. **(D)** Morphological changes of p-CA-treated HaCaT cells were observed under a phase contrast microscope. **(E)** Morphological changes of A375 and B16 cells were observed under a phase contrast microscope. Scale bar, 50 μm. **(F)** The cell colonies were stained with crystal violet and observed under an inverted microscope. All data are expressed as the mean ± SD of three independent experiments. ^&^*P* < 0.05 compared with the A375 or B16 cells after *p*-CA treatment for 24 h. *P < 0.05, **P < 0.01, ^#^*P* < 0.05, ^##^*P* < 0.01 compared with the control.

First, we used the cell counting kit-8 (CCK-8) assay to examine the effects of *p-*CA on the cell proliferation capacity. We found that *p-*CA significantly inhibited the proliferation of A375 and B16 cells (Figures 1A,B). The IC_50_ value of *p-*CA on A375 cells for 24-h treatment was 4.4 mM, and the IC_50_ value of *p*-CA for 48-h treatment was 2.5 mM (Figure 1A). Based on these IC_50_ values, we used *p-*CA concentrations of 1.5, 2.5, and 3.5 mM in subsequent A375 cell experiments. The IC_50_ value of *p*-CA on B16 cells was 4.1 mM for 24-h treatment and 2.8 mM for 48-h treatment (Figure 1B), so we used *p*-CA concentrations of 2.0, 3.0, and 4.0 mM in subsequent B16 cell experiments. At the same time, we used CCK-8 assay to examine the effect of *p*-CA on the proliferation of HaCaT cells. We found that *p*-CA significantly inhibited the proliferation of HaCaT cells at the concentration of 3.0–5.0 mM after 24 h treatment, However, the cytotoxicity of *p*-CA to HaCaT cells was significantly lower than that to A375 cells (Figures 1C,D).

Second, we observed the morphological changes of cells after 48 h of treatment with different concentrations of *p-*CA under the microscope. We clearly observed that with increasing *p-*CA concentrations, compared with the control group, the number of A375 and B16 cells decreased, the morphology changed, intercellular contact disappeared, and more cells detached (Figure 1E). Simultaneously, the results of colony formation experiments showed that *p-*CA could inhibit the colony formation of A375 and B16 cells (Figure 1F).

### *p*-CA Causes A375 and B16 Cell Cycle Arrest

**Figure 2:**
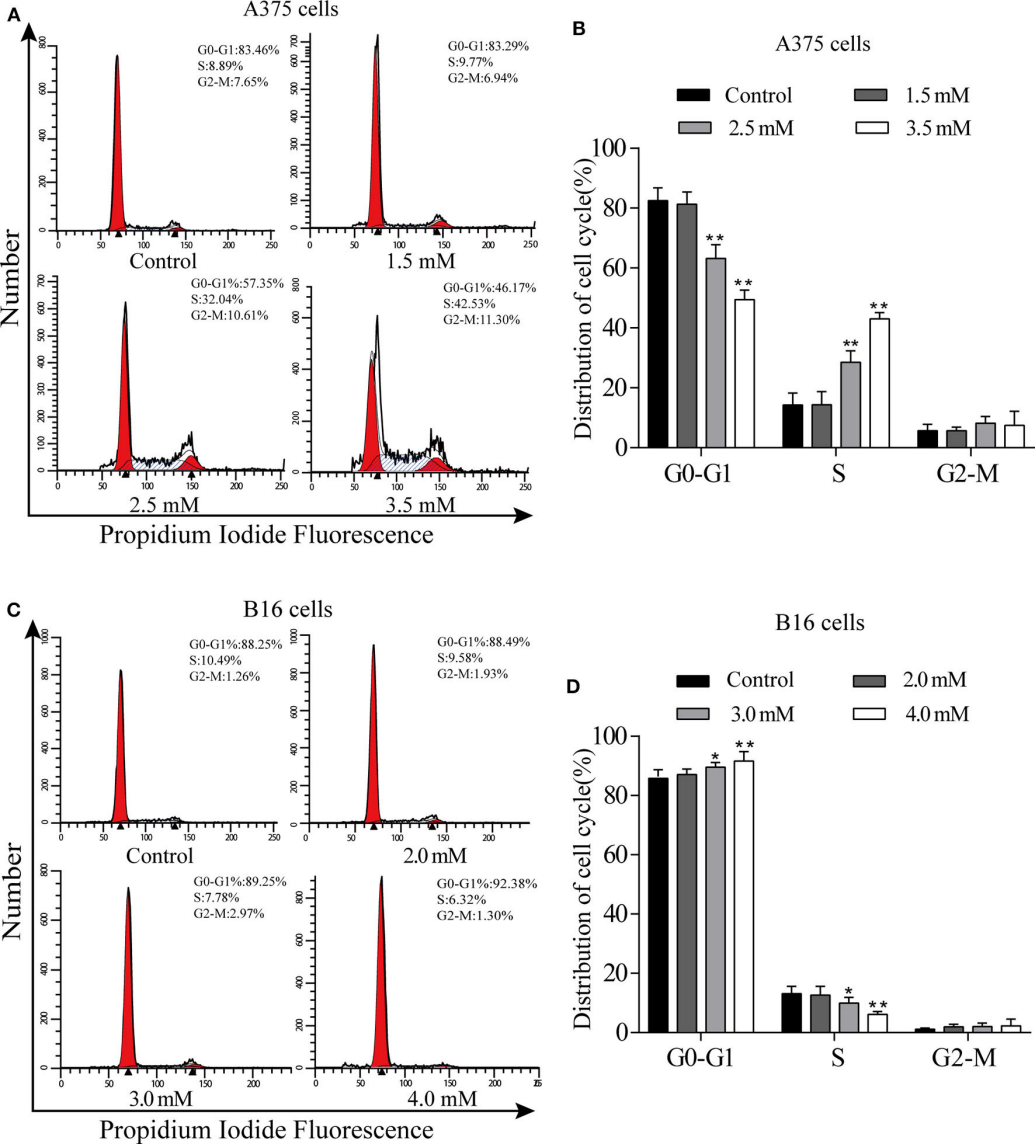
*p-*CA causes A375 and B16 cell cycle arrest. **(A)** The cell cycle distribution of A375 cells treated with *p-*CA was measured by flow cytometry. **(B)** Statistical analysis of the cell cycle distribution of A375 cells treated with *p-*CA. **(C)** The cell cycle distribution of B16 cells treated with *p-*CA was measured by flow cytometry. **(D)** Statistical analysis of the cell cycle distribution of B16 cells treated with *p-*CA. All data are expressed as the mean ± SD of three independent experiments. *P < 0.05, **P < 0.01 compared with the control.

We used flow cytometry to detect the cell cycle phases of A375 and B16 cells treated with different concentrations of *p*-CA for 24 h. The PI single staining results showed that the proportion of A375 cells in the S phase was significantly increased in the *p*-CA treatment groups (2.5 and 3.5 mM) (Figures 2A,B), and the proportion of B16 cells in the G0-G1 phase was significantly increased in the *p*-CA treatment groups (3.0 and 4.0 mM) (Figures 2C,D).

### *p-*CA Regulates the Activity of Cyclin–CDK Complexes and Cell Cycle Progression

**Figure 3:**
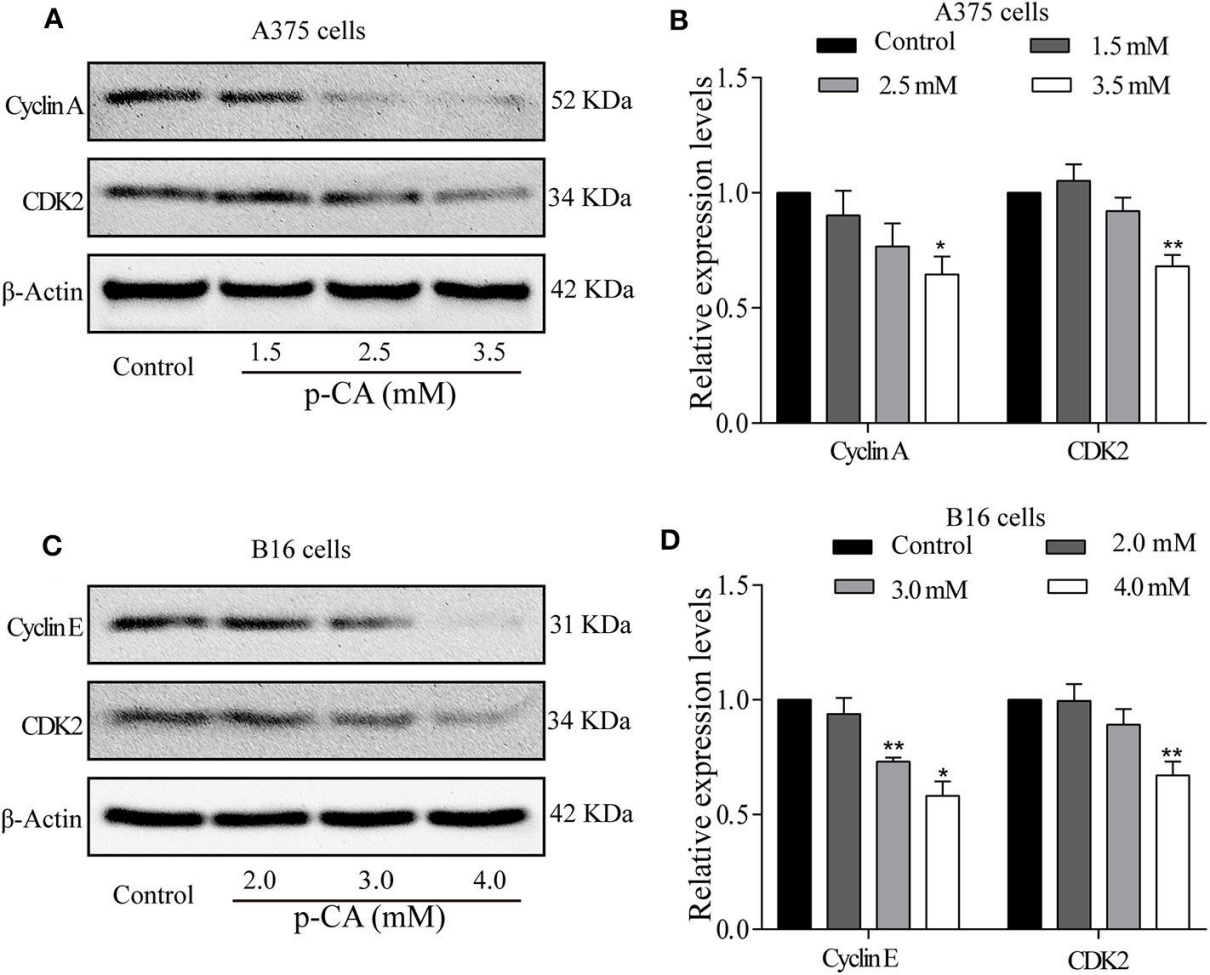
*p-*CA regulates the activity of Cyclin–CDK complexes and cell cycle progression. **(A)** The protein levels of Cyclin A and CDK2 in A375 cells treated with *p-*CA, as measured by western blot. **(B)** Statistical analysis of Cyclin A and CDK2 levels in A375 cells. The ratio of protein levels was standardized according to the value of the control. **(C)** The protein levels of Cyclin E and CDK2 in B16 cells treated with *p-*CA, as measured by western blot. **(D)** Statistical analysis of Cyclin E and CDK2 levels in B16 cells. The ratio of protein levels was standardized according to the value of the control. All data are expressed as the mean ± SD of three independent experiments. *P < 0.05, **P < 0.01 compared with the control.

To explore the mechanism by which *p*-CA changes the cell cycle, we performed western blot analysis to examine the levels of cell cycle-related proteins in *p*-CA-treated A375 and B16 cells. *p*-CA (2.5 and 3.5 mM) significantly reduced the expression levels of CDK2 and Cyclin A in A375 cells (Figures 3A,B) and the levels of CDK2 and Cyclin E in B16 cells (Figures 3C,D).

### *p-*CA Induces A375 and B16 Cell Apoptosis

**Figure 4:**
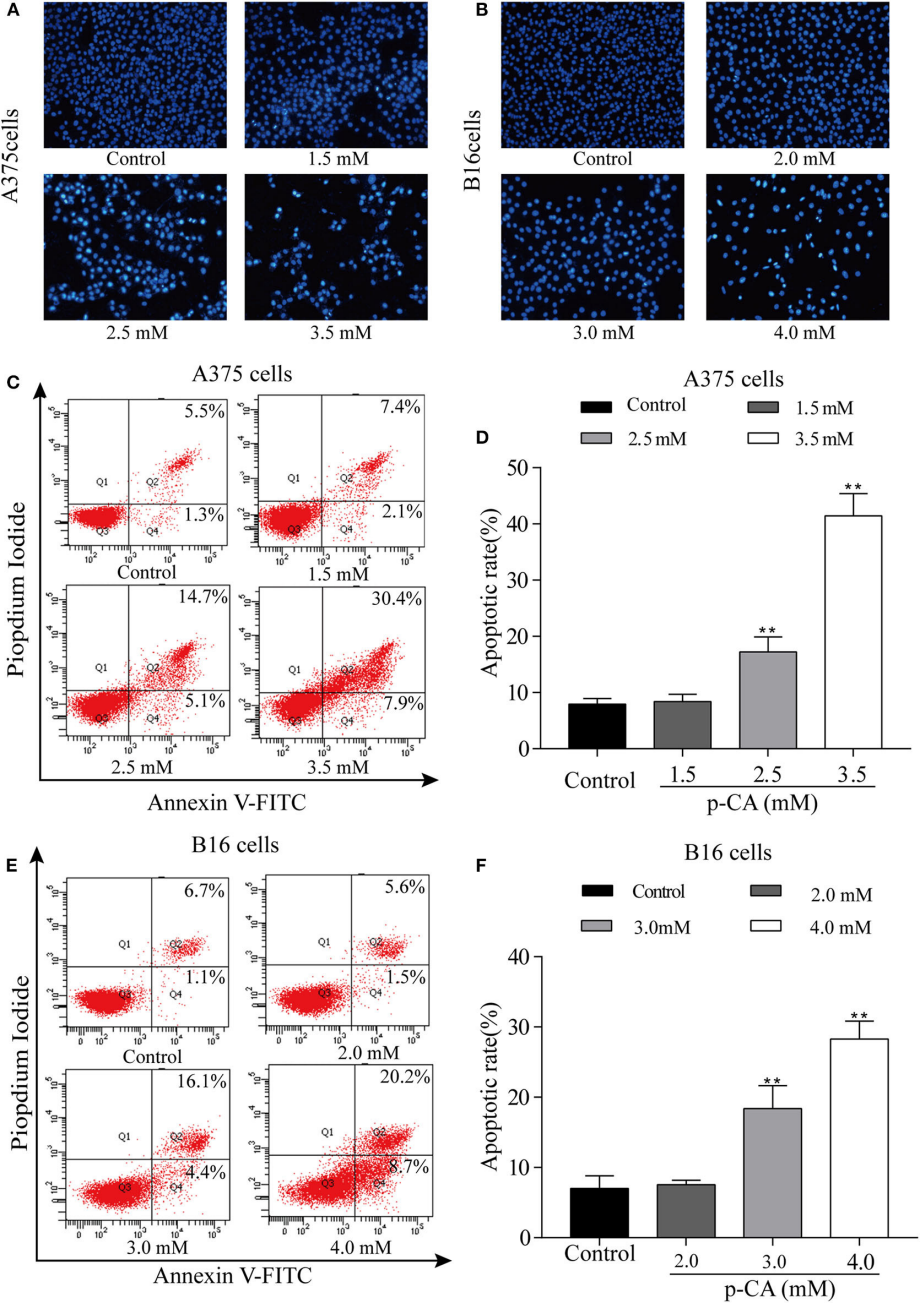
*p-*CA induces A375 and B16 cell apoptosis. **(A,B)** After Hoechst 33,258 staining, the nucleus of A375 and B16 cells treated with *p-*CA showed morphological changes typical of apoptosis. **(C)** The apoptosis rate of A375 cells treated with *p-*CA was measured by Annexin V-FITC/PI double staining. **(D)** Statistical analysis of the apoptosis rate of A375 cells treated with *p-*CA. **(E)** The apoptosis rate of B16 cells treated with *p-*CA was measured by Annexin V-FITC/PI double staining. **(F)** Statistical analysis of the apoptosis rate of B16 cells treated with *p-*CA. All data are expressed as the mean ± SD of three independent experiments. **P < 0.01 compared with the control.

The effect of *p-*CA on A375 and B16 cell apoptosis was determined by Hoechst 33,258 staining. Under normal conditions, nuclei are round and pale blue, while the fluorescence intensity of the apoptotic nucleus is higher. Moreover, apoptotic cells are more condensed and have a disturbed mass-like morphology. Apoptosis also reduces the number of cells (Figures 4A,B). We also used flow cytometry to detect the proportion of apoptotic A375 and B16 cells after treatment with *p-*CA. The results showed that the number of apoptotic cells was significantly increased upon *p-*CA treatment in both A375 and B16 cells (Figures 4C–F).

### Effect of *p-*CA on Apoptosis-Related Proteins

**Figure 5:**
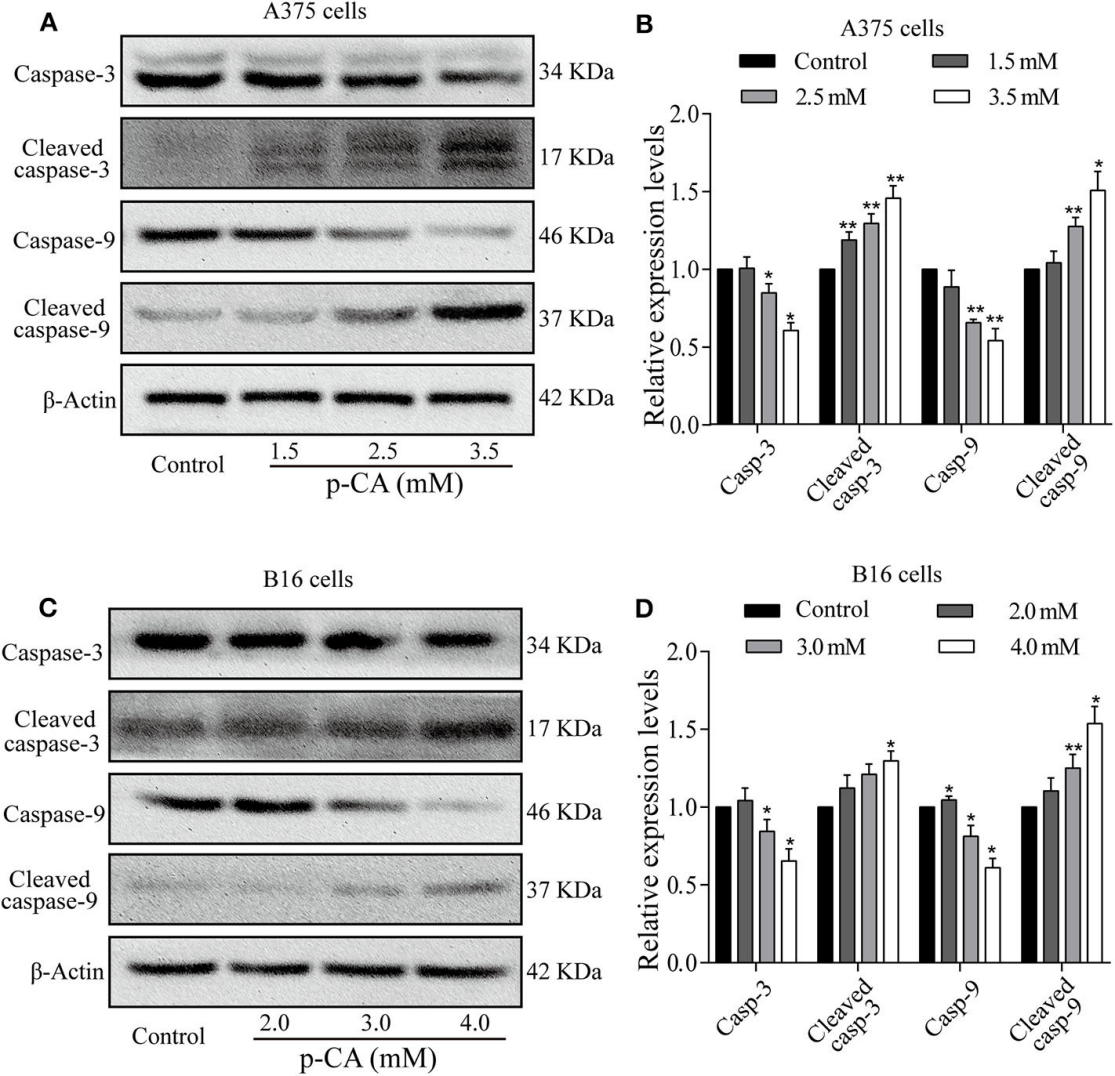
Effects of *p-*CA on apoptosis-related proteins. **(A)** The protein levels of caspase-3/9 and cleaved caspase-3/9 in A375 cells treated with *p-*CA were measured by western blot. **(B)** The relative protein levels of caspase-3/9 and cleaved caspase-3/9 in A375 cells treated with *p-*CA were statistically analyzed. **(C)** The protein levels of caspase-3/9 and cleaved caspase-3/9 in B16 cells treated with *p-*CA were measured by western blot. **(D)** The relative protein levels of caspase-3/9 and cleaved caspase-3/9 in B16 cells treated with *p-*CA were statistically analyzed. The ratio of protein levels is normalized according to the value of the control group. All data are expressed as the mean ± SD of three independent experiments. *P < 0.05, **P < 0.01 compared with the control.

To explore the mechanism of apoptosis, we performed western blot analysis to examine the levels of apoptosis-related proteins in A375 and B16 cells treated with *p*-CA for 24 h. The results showed that the levels of caspase-3 and caspase-9 decreased, and the levels of cleaved caspase-3 and cleaved caspase-9 increased in *p-*CA-treated A375 cells (2.5 and 3.5 mM) (Figures 5A,B) and B16 cells (3.0 and 4.0 mM) (Figures 5C,D). These results indicate that *p-*CA induces apoptosis in A375 and B16 cells through regulating the caspase family.

### *p-*CA Induces A375 and B16 Cell Apoptosis Through the Bcl-2/Bax Signaling Pathway

**Figure 6:**
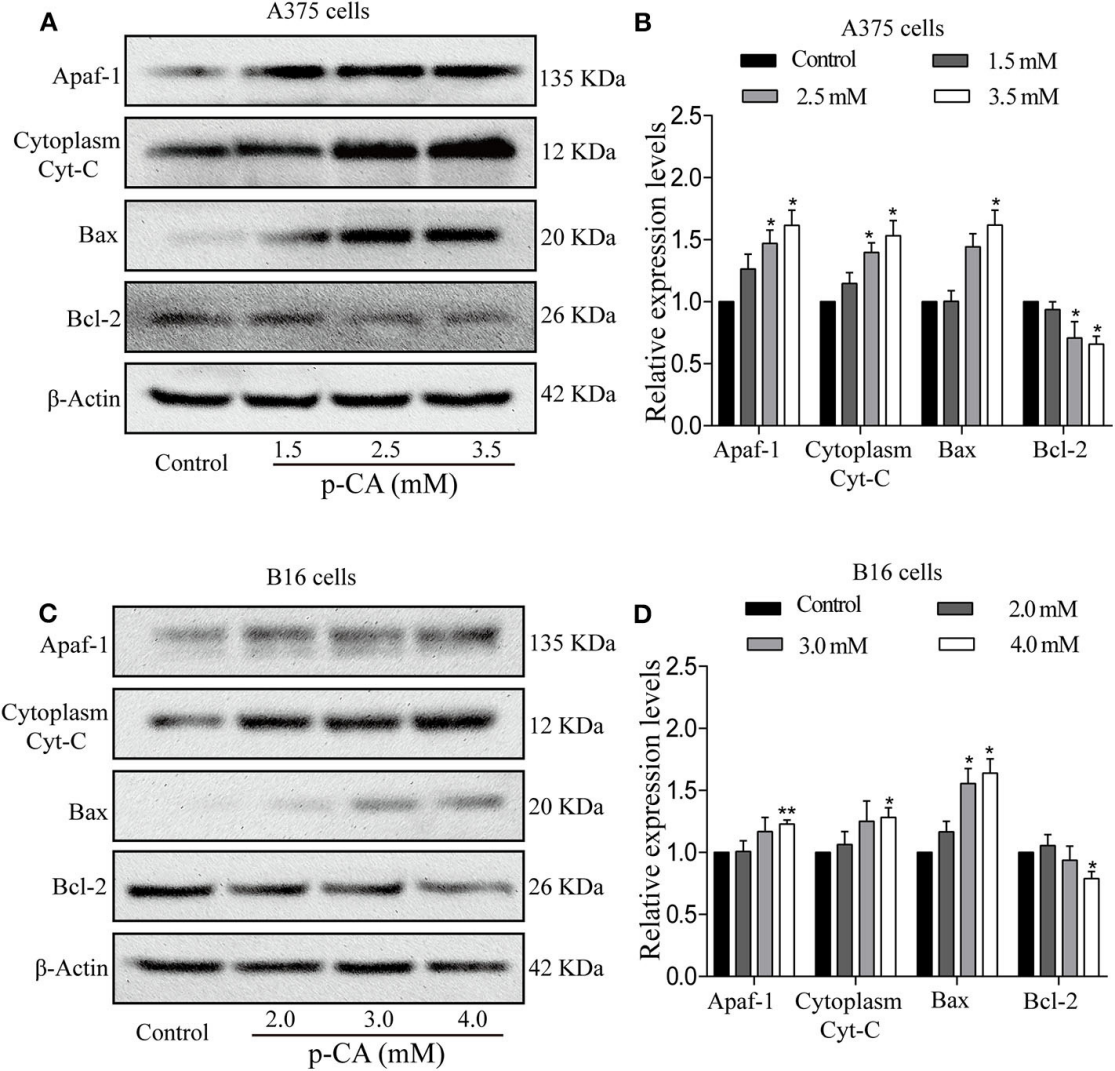
*p-*CA induces A375 and B16 cell apoptosis through the Bcl-2/Bax signaling pathway. **(A)** The protein levels of Apaf1, cytoplasmic Cyto-C, Bax, and Bcl-2 in A375 cells treated with *p-*CA were measured by western blot. **(B)** Statistical analysis of the relative protein levels of Apaf1, cytoplasmic Cyto-C, Bax, and Bcl-2 in A375 cells treated with *p-*CA. The ratio of protein levels was normalized according to the value of the control. **(C)** The protein levels of Apaf1, cytoplasmic Cyto-C, Bax, and Bcl-2 in B16 cells treated with *p-*CA were measured by western blot. **(D)** Statistical analysis of the relative protein levels of Apaf1, cytoplasmic Cyto-C, Bax, and Bcl-2 in B16 cells treated with *p-*CA. The ratio of protein levels was normalized according to the value of the control. All data are expressed as the mean ± SD of three independent experiments. *P < 0.05, **P < 0.01 compared with the corresponding control.

To further explore the underlying mechanisms, we examined the upstream regulatory factors of caspase-3 and caspase-9 in A375 and B16 cells after treatment with *p-*CA for 24 h. The results showed that *p-*CA could downregulate Bcl-2 and upregulate Bax, Apaf1, and cytoplasmic Cyto-C levels in A375 cells (Figures 6A,B) and B16 cells (Figures 6C,D).

## Discussion

*p-*CA is a phenolic acid that is widely present in fruits, vegetables, and Chinese herbal medicines (11). It has anti-inflammatory, antioxidant, anti-diabetic, anti-platelet aggregation, and anti-cancer effects. Therefore, it has the potential to be developed as an anti-tumor drug, but there have been few reports on the subject. By CCK-8 assay and colony formation experiments, we found that *p*-CA has an inhibitory effect on the proliferation of melanoma cells. According to our flow cytometry results, *p-*CA can not only promote apoptosis of melanoma cells but also change the cell cycle process. We also found the *p*-CA-induced changes in the levels of cell cycle-related and apoptosis-related proteins and upstream signaling pathway proteins.

Numerous studies have shown that tumorigenesis and cancer development are related to the disruption of the cell cycle. The cell cycle duration of tumor cells is almost the same as that of normal cells, but the proportion of actively dividing cells in tumor tissues is much higher than in normal tissues (11). The cell cycle is the entire process of cell division, which is divided into two stages: the interphase and the mitotic phase. The interphase is divided into the G1, S, and G2 phases. Cells in the G1 phase rapidly synthesize RNA and proteins, preparing materials and energy for DNA replication in the S phase. Cells in the S phase are characterized by DNA replication, doubling the DNA content. The transition from the G1 phase to the S phase is a critical moment in the cell cycle. If the cell is disturbed by certain factors, this will affect the replication of DNA, which will cause mutations or terminate DNA replication. Cells in the G2 phase are prepared for the division phase. After the interphase, the cells enter the M phase for orderly cell division (12). The cell cycle is driven by the sequential activation by Cyclins of the corresponding Cyclin-dependent kinase (CDK). CDKs play a positive role in the regulation of cell cycle progression. Cyclin-dependent kinase inhibitors (CKIs) are negative cell cycle regulators. By directly binding to CDKs, CKIs can negatively regulate CDK activity (13). Different Cyclins can specifically regulate different cell cycle stages; for example, Cyclin E and CDK2 can form a Cyclin E–CDK2 complex, allowing cells to enter the S phase from the G1 phase, and guiding cells into the G2/M phase (14). Cyclin A binds to CDK2 and is activated during the G1–S transition, thereby regulating cell proliferation (15). The cell cycle is also monitored by the cell cycle checkpoint, which is a negative feedback regulatory mechanism (16). If DNA damage is detected during the cell cycle, the cell cycle checkpoint is activated, interrupting the cell cycle process (17). Western blot results from the present study showed that *p-*CA decreases the levels of the key proteins CDK2 and Cyclin A in the A375 cell cycle, thereby blocking the progression of cells from the S to the G2 phase, increasing the proportion of S phase cells. In B16 cells, *p-*CA decreases the levels of the key proteins CDK2 and Cyclin E, causing cell cycle arrest in the G0–G1 phase. In general, *p-*CA can block proliferation of different tumor cells in different ways.

Inducing apoptosis is another effective means to inhibit tumor cell proliferation (11). Apoptosis is an orderly form of cell death (18). Cells can autonomously enter apoptosis, which involves the activation, expression, and regulation of a series of genes. The caspase and Bcl-2 families are the main proteomes that induce apoptosis (19, 20). The caspase family is the protease family that performs apoptosis (21). The caspase family can be activated to degrade or inactivate some key cellular proteins, and its activation and regulation have important significance for the transmission of apoptotic signals (22). It is believed that procaspase can be activated by three methods: self-activation, trans-activation, and non-caspase protease activation. Caspase-8 and caspase-9 can be activated by self-cleavage by binding to each other (23). Once activated, the original caspase can activate other caspase zymogens, as occurs between caspase-3 and caspase-9. In the present study, we found that *p-*CA leads to an increase in the levels of cleaved caspase-3 and cleaved caspase-9, implying that *p-*CA could induce apoptosis via a caspase-dependent mechanism.

Bcl-2 and Bax belong to the Bcl-2 family. Bcl-2 inhibits apoptosis, and Bax promotes apoptosis. They play an important regulatory role in the process of apoptosis. Studies have shown that changes in the ratio of Bcl-2 to Bax can regulate apoptosis. Bcl-2 overexpression has anti-apoptotic effects; in contrast, Bax overexpression stimulates apoptosis (24). In the present study, we found that Bcl-2 levels were significantly decreased and Bax levels were significantly increased in *p-*CA-treated A375 and B16 cells.

When cells undergo apoptosis, Bcl-2 controls mitochondrial outer membrane permeability by regulating the mitochondrial membrane potential (25). Bax, which is present in the cytoplasm, receives a signal from Bcl-2 that allows Bax to relocate to the mitochondrial surface and to form a pore across the outer mitochondrial membrane, leading to increased mitochondrial membrane permeability. Pro-apoptotic substances such as Cyto-C are released from the outer and inner membranes of mitochondria into the cytoplasm. Cytoplasmic Cyto-C interacts with Apaf1 and activates caspase-9, and activated caspase-9 can activate other caspases, such as caspase-3 and caspase-7, thereby starting the caspase cascade and causing apoptosis (25). Bal-2 levels were significantly decreased and cytoplasmic Cyto-C and Apaf1 levels were remarkably increased in *p-*CA-treated A375 and B16 cells, implying that *p-*CA could induce apoptosis in A375 and B16 cells through the mitochondrial-mediated apoptotic signaling pathway, which is regulated by the Bcl-2 family.

However, some limitations in our study should be noted. The anti-tumor effects of *p*-coumaric acid wasn't examined in a melanoma-xenografted model in nude mice. In conclusion, our results showed that *p*-CA inhibits cell proliferation in B16 and A375 cells and induces apoptosis via a mitochondrial-mediated signaling pathway. This study provides evidence for the development of *p*-CA as an anti-melanoma drug.

## Materials and Methods

### Cell Lines and Cell Culture

Mouse melanoma B16 cells (Cat. No. TCM 2) and human melanoma A375 cells (Cat. No. SCSP-533) from the Cell Bank of Type Culture Collection of Chinese Academy of Sciences China (Shanghai, China) were cultured in a sterile cell culture chamber (HF240, HEALFORCE, Shanghai Lishen Scientific Equipment Co. Ltd.) at 37°C with 95% air and 5% CO_2_ with saturated humidity. B16 cells were cultured in 1,640 medium (Cat. No. SH30809.01, Hyclone) supplemented with 10% FBS (Cat. No. 10091-148, Gibco) and 1% streptomycin solution (Cat. No. P1400, Solarbio). A375 cells were cultured in DMEM high glucose medium (Cat. No. SH30022.01, Hyclone) supplemented with 10% FBS, 1% streptomycin solution, and 1% sodium pyruvate (Cat. No. SP0100, Solarbio). Similarly, the normal human keratinocyte cell line HaCaT cells (Cat. No. KG300, KeyGEN BioTECH) were cultured in a DMEM high glucose medium supplemented with 10% FBS and 1% streptomycin solutions.

### CCK-8 Assay

Well-grown A375 cells, B16 cells and HaCaT cells were digested with 0.25% trypsin and collected by centrifugation at 800 × *g* for 3 min. Cells (100 μl) were plated in a 96-well plate (8 × 10^3^ cells/well). The cells were cultured for 24 h, and the medium was replaced with 200 μl complete medium containing *p-*CA (1.5, 2, 2.5, 3, 3.5, 4, 4.5, and 5 mM). The complete medium containing 1%0 DMSO was used as the control group. Cells were incubated for an additional 24 or 48 h, and 10 μl CCK-8 solution (Cat. No. CK04, Dojindo) was added to each well for 2 h. Finally, the absorbance of each well at 450 nm was measured by a multi-plate reader (Infinite200PRO, Tecan, Tecan Austria GmbH, Grödig, Austria).

### Colony Formation Assay

A375 and B16 cells were centrifuged at 800 × *g* for 3 min. Cells (3 ml) were plated in a 6-well plate (250 cells/well). The cells were cultured for 1 day, and the medium was replaced with 3 ml complete medium containing *p-*CA (1.5, 2.5, and 3.5 mM for A375 cells; 2.0, 3.0, and 4.0 mM for B16 cells). Cells were incubated for 48 h, and the medium was refreshed. After 14 days, the culture medium was discarded, and the cells were washed twice with PBS. Cells were fixed with 4% paraformaldehyde for 15 min, stained with 1% crystal violet (Cat. no. G1062, Solarbio) for 10 min, washed three times with PBS (until the liquid was clear), and dried at room temperature. Images were taken to observe the colony formation.

### Hoechst 33,258 Staining

The cells were digested with 0.25% trypsin and centrifuged at 800 × *g* for 3 min. Cells (2 ml) were plated in a 6-well plate (3 × 10^5^ cells/well). The cells were cultured for 24 h, and the medium was replaced with 3 ml complete medium containing *p-*CA (1.5, 2.5, and 3.5 mM for A375 cells; 2.0, 3.0, and 4.0 mM for B16 cells). Cells were incubated for 48 h and washed twice with PBS. Fixation solution (500 μl of methanol:acetic acid = 3:1) was added to each well for 10 min. Cells were washed twice with PBS, and Hoechst 33,258 staining solution (0.5 ml) was added to each well for 5 min. Cells were washed three times with PBS. Nuclear morphology was observed under an inverted fluorescence microscope.

### Annexin V-FITC/PI Flow Cytometry for Apoptosis Detection

Cells were digested with 0.25% trypsin and centrifuged at 800 × *g* for 3 min. Cells (4 ml) were inoculated in a petri dish (6 × 10^5^ cells/plate). The cells were cultured for 24 h, and the medium was replaced with 3 ml complete medium containing *p-*CA (1.5, 2.5, and 3.5 mM for A375 cells; 2.0, 3.0, and 4.0 mM for B16 cells). The cells were incubated for 48 h, trypsinized, and centrifuged at 2,000 × *g* for 3 min at 4°C. The cells were resuspended in pre-cooled PBS and centrifuged again at 2,000 × *g* for 3 min at 4°C. Annexin V-FITC and PI were added, the solutions were mixed well, and the samples were incubated for 10 min in the dark at 4°C. Cell fluorescence was detected by flow cytometry.

### Cell Cycle Detection

The cells were inoculated in a 6-well plate at a suitable concentration and cultured in a 5% CO_2_ incubator at 37°C for 24 h. The medium was replaced with complete medium containing *p-*CA (1.5, 2.5, and 3.5 mM for A375 cells; 2.0, 3.0, and 4.0 mM for B16 cells), and the cells were incubated for 48 h. The cells were digested with 0.25% trypsin, centrifuged at 800 × *g* for 3 min at 4°C, resuspended in PBS, and centrifuged again. The cell pellet was resuspended in fixation solution (70% pre-cooled ethanol) and incubated at 4°C overnight. The cells were centrifuged, RNase A solution was added to the cells, and cells were resuspended and incubated in a water bath at 37°C for 30 min. PI staining solution was added at 4°C for 30 min, and the cell cycle distribution was analyzed by flow cytometry.

### Western Blot Analysis

Cells were treated with *p-*CA (1.5, 2.5, and 3.5 mM for A375 cells; 2.0, 3.0, and 4.0 mM for B16 cells) for 24 h. The cells were collected in lysis buffer and incubated on ice for 30 min. The cells were mixed every 10 min. The cells were centrifuged at 12,000 × *g* at 4°C for 10 min. The supernatant was collected, and the protein concentration was determined. Equal amounts of protein (40 μg/slot) were subjected to 10–12% SDS-PAGE. Proteins were transferred to a PVDF membrane, which was incubated with 5% milk blocking solution for 2 h, followed by incubation with the following primary antibodies (all from Abcam, Cambridge, UK): anti-Cyclin A (1:1,000, ab53699), anti-Cyclin-dependent kinase 2 (CDK2, 1:1,000, ab32147), anti-Cyclin E (1:1,000, ab133266), anti-Bcl-2-associated X (BAX, 1:1,000, ab32503), anti-B-cell lymphoma-2 (Bcl-2, 1:1,000, ab32124), anti-cytochrome C (Cyto-C, 1:1,000, ab13575), anti-apoptotic protease activating factor-1 (Apaf1, 1:1,000, ab2000), anti-cleaved caspase-3 (1:1,000, ab32042), anti-caspase-3, (1:1,000, ab13585), anti-cleaved caspase-9 (1:1,000, ab2324), and anti-caspase-9 (1:1,000, ab202068). Subsequently, the membranes were washed with TBST buffer for 45 min and then probed with appropriate horseradish peroxidase-conjugated secondary antibodies for 1 h. Immunoreactive bands were visualized by the Novex™ ECL Chemiluminescent Substrate Reagent Kit (WP20005; Thermo Fisher Scientific, Shanghai, China) using a film processor (BioSpectrum Imaging System, Upland, CA, USA). The gray-scale value of each band was calculated by Image Pro Plus 6.0 (IPP6) software.

### Statistical Analysis

All experiments were conducted at least three times. All data are shown as the mean ± standard deviation (SD). The Student *t*-test, one-way analysis of variance (ANOVA), or two-way ANOVA was employed to analyze the statistical differences. The analyses were performed using SPSS 21.0 software (version 21.0, SPSS Inc., Chicago, IL, USA). *P* < 0.05 were considered statistically significant.

## Data Availability Statement

The raw data supporting the conclusions of this article will be made available by the authors, without undue reservation.

## Author Contributions

XH, ZY, QZ, and DL designed the study. XH, ZY, and WL acquired the data and wrote the manuscript. XH, ZY, and WL collected cell samples for Hoechst 33,258 staining, cell cycle, and western blot analyses. ZP, XZ, ML, and XL interpreted and analyzed the data. QZ and DL revised and approved the final version of the manuscript. All authors contributed to the article and approved the submitted version.

## Conflict of Interest

The authors declare that the research was conducted in the absence of any commercial or financial relationships that could be construed as a potential conflict of interest.
